# Clinical Analysis of Pediatric Opsoclonus-Myoclonus Syndrome in One of the National Children's Medical Center in China

**DOI:** 10.3389/fneur.2021.744041

**Published:** 2021-10-08

**Authors:** Haixia Zhu, Wenlin Wu, Lianfeng Chen, Chi Hou, Yiru Zeng, Yang Tian, Huiling Shen, Yuanyuan Gao, Yani Zhang, Bingwei Peng, Wen-Xiong Chen, Xiaojing Li

**Affiliations:** Department of Neurology, Guangzhou Women and Children's Medical Center, Guangzhou Medical University, Guangzhou, China

**Keywords:** opsoclonus-myoclonus syndrome, paraneoplastic syndrome, ataxia, rituximab, pediatric

## Abstract

**Objective:** To study the clinical characteristics and treatment of pediatric opsoclonus-myoclonus syndrome (OMS).

**Methods:** We analyzed the clinical data of nine children OMS between June 2017 and Nov 2020.

**Results:** Nine children (M/F = 3:6, median onset age was 18 months) diagnosed with OMS were included in the study. Before onset, human rhinovirus and respiratory syncytial virus were seen in one patient, respectively. And one patient received Japanese encephalitis vaccination. Three patients had neuroblastoma, and one patient had ganglioneuroblastoma. All patients' symptoms were improved after receiving surgery (for four patients with tumor), intravenous human immunoglobulin and pulsed methylprednisolone. However, four patients without mass relapsed and became relapse free after rituximab treatment. The relapse rate was 44.4% (4/9). The OMS severity score at the last follow-up was significantly lower than the OMS severity score at onset (3.0 ± 1.0 vs. 11.0 ± 2.2, paired-samples *t*-test, *P* < 0.001). All patients had at least one item of neurological symptoms or neuropsychological disturbances.

**Conclusion:** For pediatric OMS, human rhinovirus infection and respiratory syncytial virus infection can be seen before onset. Rituximab is effective in reducing relapse. Improving recognition and long-term prognosis in OMS is urgent.

## Introduction

Opsoclonus-myoclonus syndrome (OMS) is a rare neurological disease. The incidence of OMS was 0.18 cases per million population per year in a prospective survey of United Kingdom pediatric neurology centers and 0.27 to 0.40 cases per million in Japanese children (1, 2). It usually affects children with a median age of 18–22 months (3). The disease is characterized by acute or subacute episodes of ataxia, opsoclonus (bursts of high-frequency oscillations of the eyes with horizontal, vertical, and torsional saccades), myoclonus (non-epileptic involuntary jerks of the limbs and trunk), irritability, sleep disturbances, and behavioral disruption (3). The absence of one or some distinctive features in the early stages of the disease may delay the diagnosis of OMS by weeks or months. OMS is a rare paraneoplastic or possibly post-viral severe neurologic syndrome (3). In adults with paraneoplastic OMS, small cell lung cancer, breast carcinoma, and ovarian teratoma were the most frequently reported tumors. In contrast, more than half of pediatric OMS cases are associated with neuroblastoma (NB) (3). Although the etiology of OMS is thought to be immune mediated, disease-associated autoantibodies have yet to be identified. OMS is an important differential diagnosis of acute post-infection cerebellar ataxia, with significant differences in treatment and prognosis. About 70% of children with OMS suffer from motor, cognitive, and language impairment, so early diagnosis and timely treatment are crucial (4, 5). In order to improve recognition and determine the optimal treatment approach for children with OMS, we conducted a single-center retrospective review of children with OMS at Guangzhou Women and Children's Medical Center in China between June 2017 and Nov 2020.

## Manuscript Formatting

### Subjects and Methods

#### Subjects

Children with OMS were retrospectively recruited from June 2017 and Nov 2020 in the department of neurology of Guangzhou Women and Children's Medical Center, one of the National Children's Medical centers in China. Diagnostic criteria for OMS was defined as requiring three of the four following features (3): (1) opsoclonus, (2) myoclonus or ataxia, (3) behavioral change or sleep disturbance, and (4) NB. The relapse of OMS was defined as worsening of OMS symptoms lasting for at least 72 h after a period of stability or improvement for at least 30 days, or the escalation of immunotherapy as a proxy measure (6). This study was approved by the Ethics Committee of Guangzhou Women and Children's Medical Center. Written and signed consent was obtained from the patient's parents or guardians, who also explicitly consented to publish their personal details, clinical data and images that could identify them.

#### Methods

##### Clinical Data Collection

Clinical data, including the history of prodromal infection and vaccination, clinical manifestations, laboratory tests, neuroelectrophysiological examination, imaging data, treatments, and prognosis, were retrieved from electronic medical records and electronic medical record-assisted telephone follow-up. A standardized 0–15 point grading scale for OMS symptom severity, developed by Drs. Wendy Mitchell and Michael Pike, was used. And symptoms assessed in the scale included stance, gait, hand and arm function, opsoclonus, mood and behavior (7). The development quotient (DQ) of patients was calculated by Gesell development schedules at last follow-up visit.

##### Statistical Analysis

Statistical analysis was performed with IBM SPSS 20.0 for windows (SPSS Inc., Chicago IL, USA). Quantitative data with normal distribution were described by mean ± SD and compared by independent *t*-test or paired samples *t*-test. The correlation between two Quantitative data with normal distribution was analyzed by the Pearson correlation coefficient analysis. Categorical data were described as frequency and percentage otherwise as median with the range. *P* < 0.05 (two-sided) was considered statistically significant. Figures were graphed using GraphPad Prism 7.01 (GraphPad Software Inc., US).

### Results

#### Demographic and Clinical Characteristic Data

A total of nine children (male: female was 3:6) diagnosed with OMS were involved. The median age of symptom onset was 18 months (ranged from 10 to 20 months), while the median age of diagnosis was 22 months (ranged from 13 to 35 months). All patients normally developed before the onset of OMS. At the initial evaluation in our hospital, opsoclonus, myoclonus, and ataxia were seen in all nine patients. Five patients had sleep disturbances (#2,3,5,8,9), seven patients (#1,2,3,4,7,8,9) displayed irritability and five patients (#3,5,7,8,9) exhibited speech disorders. The OMS severity score at the initial evaluation in our hospital was 11 ± 2.2. During the whole disease course, ataxia, opsoclonus, myoclonus, motor and sleep disturbances were seen in all patients, while irritation (#1,2,3,4,5,7,8,9) and speech disorders (#1,2,3,5,6,7,8,9) were found in eight patients.

#### Prodromal Events

Seven patients (#1,2,3,4,5,8,9) had prodromal events 1 week before symptom onset. Six of them had upper respiratory infection disease (#1,3,4,5,8,9), and one patient (#2) had vomiting and diarrhea, while this patient also received an anti-Japanese encephalitis vaccination 1 week before onset.

#### Ancillary Test Results

##### Peripheral Blood Test

Blood lactic acid/pyruvic acid, blood ammonia, blood urine organic acids, amino acids and blood acylcarnitine were tested normal in all patients.

##### Pathogenic Examination

Seven patients (#1,2,3,4,6,7,9) were tested for human chlamydia psittaci, mycoplasma pneumonia, respiratory adenovirus, enterovirus, influenza A, influenza B, human rhinovirus, respiratory syncytial virus with throat swab samples. Patient #1's was positive for human rhinovirus and patient #2's was positive for respiratory syncytial virus. Cerebrospinal fluid (CSF) was tested negative for the nucleic acid of enterovirus, influenza A, influenza B, Herpes simplex virus, Epstein-Barr virus, Cytomegalovirus and antibodies of human chlamydia psittaci and mycoplasma pneumonia in all patients.

#### Screening for Tumors and Tumor Characteristics

##### Tumor Markers

All patients underwent tumor markers tests, including 24-h urinary vanillylmandelic acid, serum neuron enolase, alpha-fetoprotein and other examinations. All findings are within normal ranges, except mild increase of serum neuron enolase in three patients (#1, 2, 4).

##### Tumor Detection

All patients underwent chest and abdomen computed tomography for tumor detection. Chest mass was in three patients (#1,4,6), while abdominal mass was found in two patients (#5,9). Four (#1, 4, 6, 9) of these five patients underwent bone marrow examination through bone marrow puncture, and the results were normal. These four patients received tumor removal surgical treatment. According to the pathology results of the tumor, three patients (#1,4,9) were diagnosed as NB (all their tumor were at stage 2 assessed by international NB staging system) and one patient (#6) was diagnosed as ganglioneuroblastoma (stage 1 assessed by international NB staging system, more details seen in Table 1). One patient (#5) declined surgery treatment.

**Table 1:** Clinical and histological characteristics of NB patients with OMS.

**No./sex**	**Age at NB** **diagnosis (months)**	**Tumor localization**	**INSS**	**Histology**	***MYCN* status**	**NSE (ng/ml)**	**Urine VMA (mg/d)**	**Bone marrow examination**
1/M	19	Left-sided paravertebral	2	NB	Non-amplified	28.35	<2	Normal
4/M	13	Right-sided paravertebral	2	NB	Non-amplified	22.66	<2	Normal
6/F	35	Right-sided paravertebral	1	Ganglio-neuroblastoma	Non-amplified	27.22	<2	Normal
9/M	24	Right adrenal gland	2	NB	Non-amplified	21.52	3.5	Normal

*INSS, International NB Staging System; NB, Neuroblastoma; NES, neuron enolase; OMS, opsoclonus-myoclonus syndrome; VMA, vanillylmandelic acid*.

#### Brain Magnetic Resonance Image and Electroencephalography

All patients underwent brain magnetic resonance image and electroencephalography examination. Eight patients showed no abnormalities in brain magnetic resonance image and one patient (#1) showed the lateral ventricle slightly enlarged. Electroencephalography in two patients (#7, 9) showed non-specific slow wave background and electroencephalography in the others were normal.

#### Treatment

**Figure 1:**
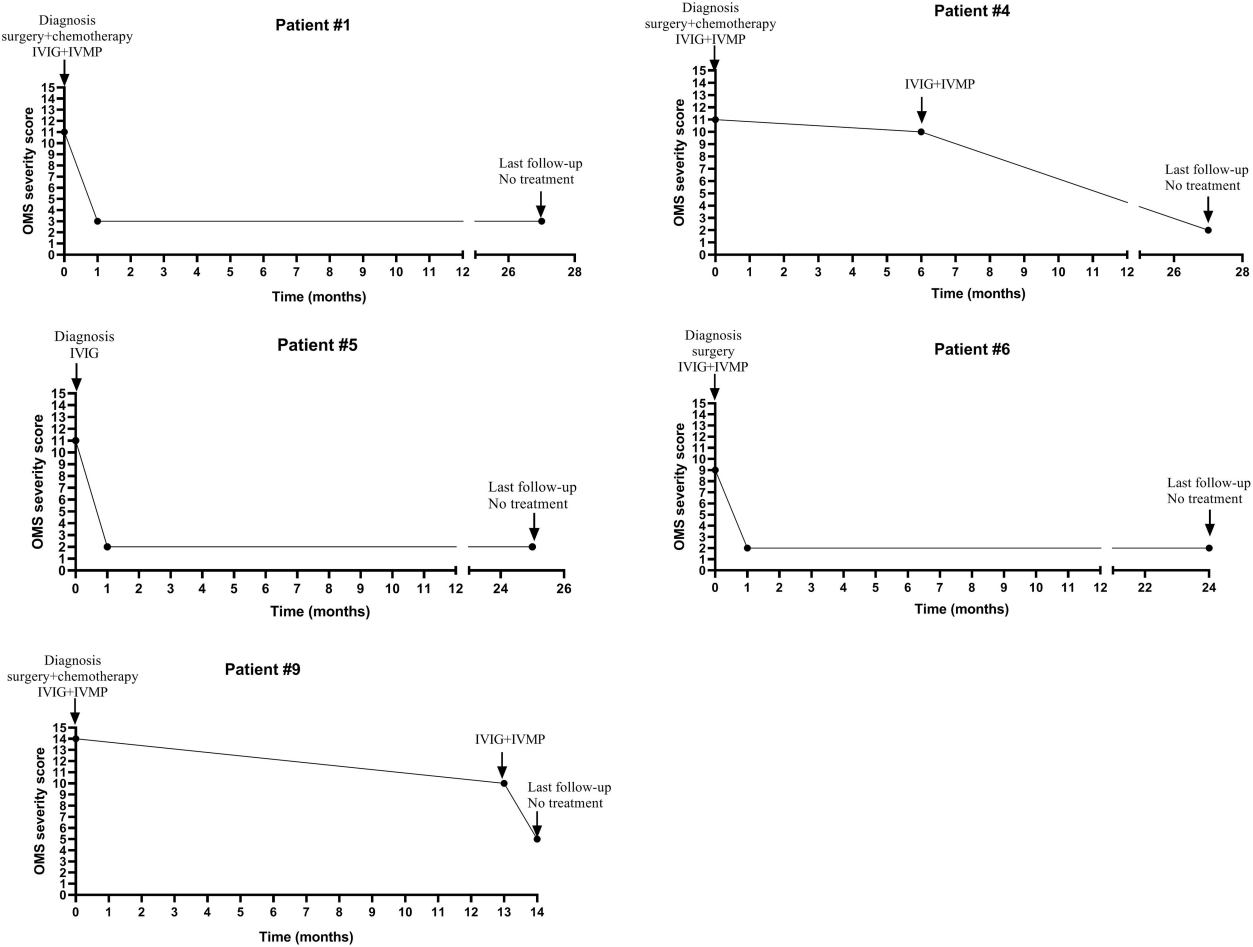
The change of OMS severity score and immunotherapy of five patients with tumor or mass during the clinical course.

Four patients (#1,4,6,9) with tumor were treated with surgery, intravenous human immunoglobulin (IVIG) (a dose of 2 g/kg over a period of 2–5 days) combined with intravenous pulsed methylprednisolone (IVMP, 10–15 mg/kg/day infusion for 3 days) and chemotherapy (for patient #1,4,9). Their Symptoms were improved without relapse and OMS severity score decreased (details seen in Table 2; Figure 1). One patient (#5) with mass declined surgery treatment and only received IVIG treatment (a dose of 2 g/kg over a period of 5 days). Her OMS scores decreased from 11 to 2 in the last follow-up (details seen in Table 2; Figure 1).

**Table 2:** Clinical features, treatment, and prognosis of children with OMS (*N* = 9).

**No./Sex**	**Onset age (months)**	**Diagnosis age (months)**	**Tumor**	**Prodromal infection**	**OMS severity score at onset**	**RTX treatment**	**Time interval^a^ (months)**	**Number of relapses**	**OMS severity score at last follow-up**	**Duration of follow-up (months)**	**DQ at last follow-up**
1/M	14	19	+	+	11	NO	1	0	3	27	94
2/F	10	16	–	+	14	YES	1	3	4	28	81
3/F	18	19	–	+	11	YES	1	3	2	36	74
4/M	12	13	+	+	11	NO	6	0	3	27	80
5/F	20	22	+/–	+	8	NO	1	0	2	25	76
6/F	18	35	+	–	9	NO	1	0	2	24	69
7/F	30	30	–	+	9	YES	1	2	3	17	90
8/F	21	25	–	+	13	YES	1	1	3	10	69
9/M	20	24	+	+	14	NO	13^b^	0	5	14	65

*DQ, development quotient; F, female; M, male; OMS, opsoclonus-myoclonus syndrome; RTX, Rituximab; IVIG, intravenous immunoglobulin; IVMP, intravenous methylprednisolone*.

a *Time interval: Time from diagnosis to initial OMS symptom improvement*.

b *This patient delayed revisiting to our hospital 10 months after chemotherapy for fearing Covid-19*.

**Figure 2:**
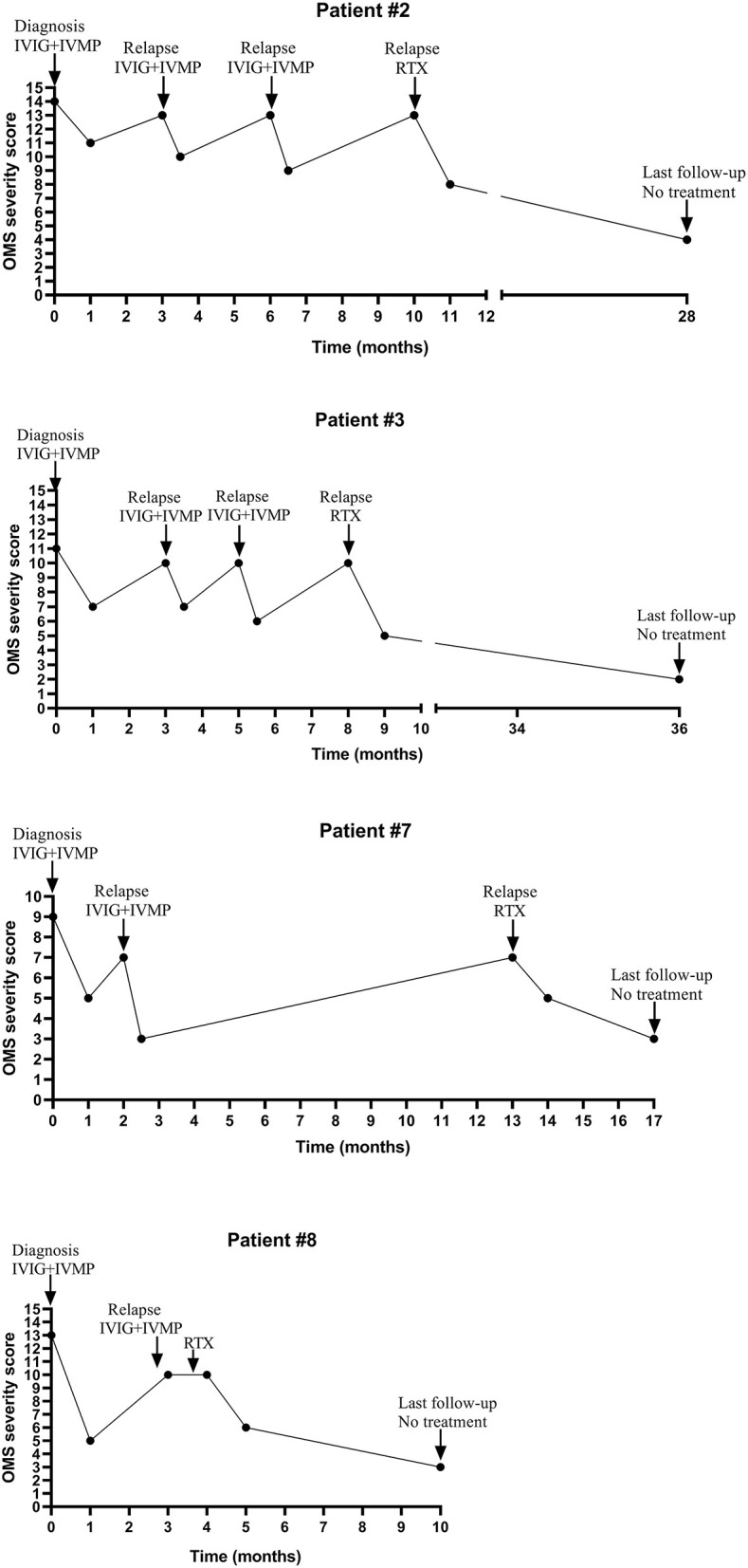
The change of OMS severity score and immunotherapy of four relapsed patients during the clinical course.

Four patients (#2,3,7,8) without tumor or mass received initial IVIG and IVMP treatment tapered off within 6 months. Symptoms in these four patients were improved, but they had several relapses during the weaning off steroids (the dose of prednisone ranged from 0.42 to 1.25 mg/kg/d when relapsed). During relapses, IVIG combined with IVMP treatment could still improve OMS symptoms. These four patients experienced one to the three times relapses, so they received Rituximab (RTX) treatment (375 mg/m^2^ weekly for 4 weeks). After RTX treatment, the patients' condition was improved and they did not relapse during a follow-up ranging from 6 to 29 months (more details seen in Table 2; Figure 2).

#### Course and Relapse

The median interval between onset and diagnosis was 4.5 months (ranged from 4 weeks to 17 months). The relapse rate was 44.4% (4/9). The relapse rate in patients with tumor or mass was 0%, while in patients without tumor or mass was 100% (more details seen in Table 2; Figure 1).

#### Follow-Up

Up to Nov 2020, none was lost to follow-up. The median follow-up interval was 25 months (ranging from 10 to 36 months). The OMS severity score at the last follow-up was significantly lower than the OMS severity score at onset (3.0 ± 1.0 vs. 11.0 ± 2.2, paired-samples *t*-test, *P* < 0.001). The DQ of patients at the last follow-up was 77.6 ± 9.8 (ranged from 65 to 94, more details seen in Table 2). The DQ of patients at the last follow-up was not correlated with the age at onset, the OMS severity score of onset or last follow-up, the number of relapses, the duration of follow-up (*P* > 0.05, Pearson correlation coefficient analysis). There was no statistical difference in the DQ at the last follow-up between patients with tumor or not (76.8 ± 11.3 vs. 78.5 ± 9.1, *t* = 0.244, independent *t*-test, *P* = 0.814). There was no statistically difference in the DQ at the last follow-up between patients receiving RTX treatment or not (78.5 ± 9.1 vs. 76.8 ± 11.3, *t* = −0.244, independent *t*-test, *P* = 0.814). All patients had at least one item of neurological symptoms or neuropsychological disturbances at the most recent evaluation. Eight patients (88.9%) had delayed speech development, typically present with lack of vocabulary. Five patients (55.6%) had mild ataxia, 2 (22.2%) had mild opsoclonus, and 3 (33.3%) had behavioral disorders characterized by irritability, hyperactivity, and restlessness in sleep. None of the children with NB had tumor recurrence during the follow-up period.

### Discussion

Our study described nine pediatric patients with OMS retrospectively. Their median age at onset was 18 months (ranged from 10 to 20 months), similar to the onset age in other reports, ranging from 6 months to 4 years (2, 5, 8–13). The ratio of male to female is 3:6, which was also consistent with other reports showing OMS with a female predominance (8, 9).

Though Marcel Kinsbourne first described OMS in 1962, the pathogenesis of OMS is still unclear. Before OMS onset, some patients could have infectious events and a few children with NB could develop OMS. In Pranzatelli et al.'s study, 31% of patients had prodromal symptoms of a possible non-specific upper respiratory illness, vomiting in 26%, diarrhea in 14%. In our study, six patients had symptoms of upper respiratory tract infection, and one patient had both vomiting and diarrhea. In many case reports, the virus was the most common infectious seen in OMS, including west-Nile virus (14, 15), human herpes virus-6 (16, 17), Epstein-Barr virus (18), adenovirus (19), influenza A virus (20), varicella-zoster virus (21), rotavirus (22) and herpes simplex virus (23). OMS onset followed mycoplasma pneumonia has also been reported (24–27). In our study, two patients had rhinovirus infection and respiratory syncytial virus infection, respectively. Rhinovirus infection and respiratory syncytial virus infection were not reported in previous studies. Viral illnesses are prevalent among patients and healthy children at the age of OMS onset. Whether these infections are occasionally presented in OMS or play a role in the pathogenesis of OMS needs further investigation. Moreover, it is also important to note that the presence of infection does not exclude a NB (as patient #1, 4, and 9 in our study), and therefore one always needs to look for NB. Interestingly, one female patient in our study received Japanese encephalitis vaccination before OMS onset. OMS onset followed by receiving vaccination was rare. In previous studies, two OMS female patients (one was 11 years old and the other one was 30 years old) received human papilloma virus vaccination and anti-Rubella vaccination about 2 weeks before OMS onset (28, 29). Japanese encephalitis vaccination was associated with some autoimmune diseases in the central nervous system like anti-N-Methy-D-aspartate receptor encephalitis and acute disseminated encephalomyelitis (30). Though there was no direct evidence about how the Japanese encephalitis vaccination triggered these autoimmune diseases, Wang et al. found some miRNAs associated with the Japanese encephalitis vaccination, were closer to let-7 family which was related to anti-N-Methy-D-aspartate receptor encephalitis by phylogenetic tree analysis (30). And Wang et al.'s study also provided a protocol for investigating the association of vaccination and anti-N-Methy-D-aspartate receptor encephalitis. This method may be helpful to study the association of infection or vaccination with OMS in further study.

The classic clinical manifestations of OMS are opsoclonus (conjugation, non-phasic, fast and multi-direction), myoclonus (non-epileptic limb convulsions), ataxia, sleep disturbances, cognitive dysfunction, and behavioral (3). The earlier appearance of ataxia than opsoclonus or myoclonus in OMS may provide one reason why acute cerebellar ataxia is such a diagnostic pitfall. In Pranzatelli et al.'s studies, the interval between OMS onset and diagnosis was 2.8 months in 2005, while it was 1.2 months in 2017. In Mitchell et al.'s study, the interval between OMS onset and diagnosis ranged from 2 days to 14 months. In our study, this interval ranged from 1 to 17 months, with a median interval of 3 months. Evidence suggests that at least 20% of OMS cases have an atypical presentation. For example, opsoclonus may occur 1–18 months after onset, so the diagnosis is often delayed by weeks or months (31). Opsoclonus is a vital symptom to distinguish OMS from acute ataxia. Though some of the patients in our study presented ataxia, myoclonus, and opsoclonus at first, were misdiagnosed by their primary care doctors as acute cerebellar ataxia before admission to our hospital. These might be caused by neglecting the opsoclonus symptom, or the OMS was a rare disorder and not be well-known by doctors who were not neurologist. OMS was rarely resolved spontaneously without immunotherapy (acute cerebellar ataxia does). For patients with ataxia presentation, if opsoclonus is observed, OMS diagnosis should be taken into consideration.

Some of the OMS is associated with malignancies. In adults, OMS is primarily associated with breast cancer and lung cancer. In children, OMS is associated in more than half of cases with NB (3). Interestingly, for reasons as yet unknown, the survival rate of children with OMS and NB is far better than that of the general population of children with NB. NB with OMS symptoms has a lower grade and no MYCN oncogene amplification and tends to have a better prognosis. OMS-associated NB is typical of low risk and carries an excellent prognosis. Low-grade tumors are often metabolically inactive, resulting in an increased urinary catecholamine uncommon. Increased vanillylmandelic acid levels can be detected only in 21% of OMS children with NB (11). In this study, four children with OMS diagnosed with NB showed normal urinary vanillylmandelic acid and no amplification of MYCN oncogene in the tumor tissues, similar to the results reported by Brunklaus et al. (32). Autoimmune pathophysiology is thought to be possible in OMS disease. And patients appear to respond to immunosuppressive treatment such as IVIG, methylprednisolone, dexamethasone, adrenocorticotropic hormone, RTX and cyclophosphamide pulses. International shared guidelines stating the optimal combination and duration of different immunomodulatory drugs are still lacking. In the beginning, the treatment protocol for OMS in our center was that when patients still relapsed after three cycles of IVIG combined with IVMP, then RTX treatment should be considered (#2, 3). Later, based on the good response of RTX treatment in case 2 and case 3, and more international studies have suggested that the effect of RTX in OMS, especially when given early in the disease course, improved OMS outcome. So we advanced the time of initiating RTX treatment when the patients still relapsed after two cycles of IVIG combined with IVMP treatment (#7, 8). In our study, neurological symptoms in patients with or without tumors were improved after treatment with IVIG combined with IVMP or IVIG only (2, 5, 9–13) (More details seen in Supplementary Material). Moreover, a randomized, open-label, phase 3 trial study from de Alarcon et al. showed that even for children with OMS associated with neuroblastoma, the addition of IVIG to prednisone and risk-adapted chemotherapy improved treatment response rate significantly (33). However, some patients would experience relapse. In our study, all patients without tumor or mass experienced relapse during weaning of steroid. This result was similar to previous studies (34). After treatment with RTX, all these four patients without tumor or mass in our study became relapse free. RTX is an anti-CD20 monoclonal antibody that depletes circulating B cells. Pranzatelli et al. found that the CD19+ B cell recruitment in the CSF of the OMS patients was correlated with their neurological severity (35). Besides, some autoantibodies were found in OMS patients, such as anti-Hu autoantibody, anti-Ru and so on and B-cell activating factor BAFF increased in the CSF in childhood OMS (34). This evidence suggested the B-cell has participated in the autoimmune pathophysiology of OMS. Furthermore, RTX effectively reduced relapse in our study and suggested this relapse might be B-cell associated. The first successful treatment of pediatric OMS patients using RTX was done by Pranzatelli et al. in 2005 (36). Subsequently, other studies also confirm the beneficial effect of RTX in OMS patients, even in otherwise treatment refractory patients or in relapsed patients (8, 9, 34). Consistently, we found RTX was effective in preventing relapse in OMS patients.

Though neurological symptoms in patients with OMS show at least some improvement under current treatment, neuropsychological disturbances became a more considerable part of the clinical course at long-term follow-up. Over 70% of OMS patients have neurological sequelae, especially intellectual disability and language developmental delay (2, 4, 5, 9–13, 34, 37) (More details seen in Supplementary Material). Similarly, in our study, eight patients (88.9%) had delayed speech development, and three patients (33.3%) had behavioral disorders characterized by irritability, hyperactivity and restlessness in sleep. We found that the DQ of patients at the last follow-up was 77.6 ± 9.8. And three OMS patients in En Lin et al.'s study had evolving cognitive dysfunction despite being in remission and been followed up for a long time (ranged from 5 to 10 years) (38). In our study, we found the DQ of patients at the last follow-up was not correlated with the age at onset, the OMS severity score onset or last follow-up, the number of relapses, the duration of follow-up. In addition, there was no statistical difference in the DQ at the last follow-up between patients with tumor or not, as well as patients receiving RTX treatment or not. A recent study conducted by Sheridan et al. showed that the number of relapses negatively correlates with full-scale IQ in pediatric OMS (6). We failed to found the correlation between the relapse and DQ in our patients might be caused by the small number of patients in our study. More active immunoregulatory strategies to reduce OMS relapses may improve the prognosis. In Michell et al.'s study from Children's Hospital Los Angeles, RTX treatment was initiated after one or more relapses for OMS patients. More recently, it became a part of initial therapy. Their study found that using such a more aggressive immunosuppression treatment improved adaptive behavior, cognitive and motor scores. Although this study did not meet the standards of a randomized clinical trial, it suggested that more aggressive treatment with RTX might be a possible way to achieve the aim that is not only the improvement of neurological symptoms but also the improvement of neuropsychological disturbances. Further study with a large-scale clinical trial is needed to investigate whether a more aggressive immunosuppression treatment can improve neuropsychological disturbances.

Our study has several limitations. The first limitation is that we were unable to test the lymphocyte subset in the CSF. Neurological severity of OMS was positively related to the percentage of CD19+ B lymphocytes in CSF and was negatively related to CD4+ T lymphocytes in CSF (35, 39). The second limitation is that our study is a retrospective study, and we did not test the oligoclonal bands in CSF which was also a marker of disease activity (40).

## Conclusions

For pediatric OMS, human rhinovirus infection and respiratory syncytial virus infection can be seen before onset. Rituximab is effective in reducing relapse. Improving recognition and long-term prognosis in OMS is urgent.

## Data Availability Statement

The original contributions presented in the study are included in the article/Supplementary Material, further inquiries can be directed to the corresponding authors.

## Ethics Statement

The studies involving human participants were reviewed and approved by the Ethics Committee of Guangzhou Women and Children's Medical Center. Written informed consent to participate in this study was provided by the participants' legal guardian/next of kin.

## Author Contributions

HZ, WW, and LC study concept, acquisition of data, and draft the manuscript. CH, YZe, YT, HS, YG, and YZh study concept and acquisition of data. BP analyzed the data. XL and W-XC study concept and critical revision of manuscript for intellectual content. All authors gave final approval of the version to be published.

## Funding

The present study was supported by the Health and family planning technological project foundation of Guangzhou city (Grant No. 20181A011038). The funders had no role in the study concept, study design, data analysis, interpretation or reporting of the results. The authors had full control of the data and information submitted for publication.

## Conflict of Interest

The authors declare that the research was conducted in the absence of any commercial or financial relationships that could be construed as a potential conflict of interest.

## Publisher's Note

All claims expressed in this article are solely those of the authors and do not necessarily represent those of their affiliated organizations, or those of the publisher, the editors and the reviewers. Any product that may be evaluated in this article, or claim that may be made by its manufacturer, is not guaranteed or endorsed by the publisher.

## Abbreviations

CSF: cerebrospinal fluid
DQ: development quotient
IVIG: intravenous human immunoglobulin
IVMP: intravenous pulsed methylprednisolone
NB: neuroblastoma
OMS: opsoclonus-myoclonus syndrome
RTX: Rituximab.

## References

[1] Pang KK de Sousa C Lang B Pike MG. A prospective study of the presentation and management of dancing eye syndrome/opsoclonus-myoclonus syndrome in the United Kingdom. Eur J Paediatr Neurol. (2010) 14:156–61. 10.1016/j.ejpn.2009.03.002 19423368

[2] Hasegawa S Matsushige T Kajimoto M Inoue H Momonaka H Oka M. A nationwide survey of opsoclonus-myoclonus syndrome in Japanese children. Brain Dev. (2015) 37:656–60. 10.1016/j.braindev.2014.10.010 25454391

[3] Matthay KK Blaes F Hero B Plantaz D De Alarcon P Mitchell WG. Opsoclonus myoclonus syndrome in neuroblastoma a report from a workshop on the dancing eyes syndrome at the advances in neuroblastoma meeting in Genoa, Italy, 2004. Cancer Lett. (2005) 228:275–82. 10.1016/j.canlet.2005.01.051 15922508

[4] Catsman-Berrevoets CE Aarsen FK van Hemsbergen ML van Noesel MM Hakvoort-Cammel FG van den Heuvel-Eibrink MM. Improvement of neurological status and quality of life in children with opsoclonus myoclonus syndrome at long-term follow-up. Pediatr Blood Cancer. (2009) 53:1048–53. 10.1002/pbc.22226 19672966

[5] Brunklaus A Pohl K Zuberi SM de Sousa C. Outcome and prognostic features in opsoclonus-myoclonus syndrome from infancy to adult life. Pediatrics. (2011) 128:e388–94. 10.1542/peds.2010-3114 21788225

[6] Sheridan A Kapur K Pinard F Dietrich Alber F Camposano S Pike MG. IQ predictors in pediatric opsoclonus myoclonus syndrome. A large international cohort study. Dev Med Child Neurol. (2020) 62:1444–9. 10.1111/dmcn.14628 32696984

[7] Gorman MP. Update on diagnosis, treatment, prognosis in opsoclonus-myoclonus-ataxia syndrome. Curr Opin Pediatr. (2010) 22:745–50. 10.1097/MOP.0b013e32833fde3f 20871403

[8] Mitchell WG Wooten AA O'Neil SH Rodriguez JG Cruz RE Wittern R. Effect of increased immunosuppression on developmental outcome of Opsoclonus Myoclonus Syndrome (OMS). J Child Neurol. (2015) 30:976–82. 10.1177/0883073814549581 25342308

[9] Pranzatelli MR Tate ED McGee NDemographic R. Clinical immunologic features of 389 children with opsoclonus-myoclonus syndrome: a cross-sectional study. Front Neurol. (2017) 8:468. 10.3389/fneur.2017.00468 28959231 PMC5604058

[10] Tate ED Allison TJ Pranzatelli MR Verhulst SJ. Neuroepidemiologic trends in 105 US cases of pediatric opsoclonus-myoclonus syndrome. J Pediatr Oncol Nurs. (2005) 22:8–19. 10.1177/1043454204272560 15574722

[11] Krug P Schleiermacher G Michon J Valteau-Couanet D Brisse H Peuchmaur M. Opsoclonus-myoclonus in children associated or not with neuroblastoma. Eur J Paediatr Neurol. (2010) 14:400–9. 10.1016/j.ejpn.2009.12.005 20110181

[12] Tate ED Pranzatelli MR Verhulst SJ Markwell SJ Franz DN Graf WD. Active comparator-controlled, rater-blinded study of corticotropin-based immunotherapies for opsoclonus-myoclonus syndrome. J Child Neurol. (2012) 27:875–84. 10.1177/0883073811428816 22378659

[13] Dale RC Brilot F Duffy LV Twilt M Waldman AT Narula S. Utility safety of rituximab in pediatric autoimmune inflammatory CNS disease. Neurology. (2014) 83:142–50. 10.1212/WNL.0000000000000570 24920861 PMC4117174

[14] Radu RA Terecoasă EO Ene A Băjenaru OA Tiu C. Opsoclonus-myoclonus syndrome associated with west-nile virus infection: case report and review of the literature. Front Neurol. (2018) 9:864. 10.3389/fneur.2018.00864 30386288 PMC6198716

[15] Hébert J Armstrong D Daneman N Jain JD Perry J. Adult-onset opsoclonus-myoclonus syndrome due to West Nile Virus treated with intravenous immunoglobulin. J Neurovirol. (2017) 23:158–9. 10.1007/s13365-016-0470-3 27473195

[16] Simon T Cheuret E Fiedler L Mengelle C Baudou E Deiva K. Acute transverse myelitis following an opsoclonus-myoclonus syndrome: an unusual presentation. Eur J Paediatr Neurol. (2018) 22:878–81. 10.1016/j.ejpn.2018.05.002 29773357

[17] Belcastro V Piola M Binda S Santoro D Rezzonico M Arnaboldi M. Opsoclonus-myoclonus syndrome associated with human herpes virus-6 rhomboencephalitis. J Neurol Sci. (2014) 341:165–6. 10.1016/j.jns.2014.04.013 24793510

[18] Verma A Brozman B. Opsoclonus-myoclonus syndrome following Epstein-Barr virus infection. Neurology. (2002) 58:1131–2. 10.1212/WNL.58.7.1131 11940712

[19] Syrbe S Merkenschlager A Bernhard MK Grosche J Liebert UG W. Hirsch. Opsoclonus-myoclonus syndrome after adenovirus infection. Springerplus. (2015) 4:636. 10.1186/s40064-015-1429-1 26543770 PMC4628014

[20] Morita A Ishihara M Kamei S Ishikawa H. Opsoclonus-myoclonus syndrome following influenza a infection. Intern Med. (2012) 51:2429–31. 10.2169/internalmedicine.51.7627 22975562

[21] Singh D Sinha M Kumar R Shukla R Ahuja RC. Opsoclonus-myoclonus syndrome caused by varicella-zoster virus. Ann Indian Acad Neurol. (2010) 13:211–2. 10.4103/0972-2327.70876 21085535 PMC2981762

[22] Gurkas E Gucuyener K Yilmaz U Havali C Demir E. Opsoclonus-myoclonus syndrome following rotavirus gastroenteritis. Pediatr Int. (2014) 56:e86–7. 10.1111/ped.12433 25521990

[23] Chen Y Chen D Zhou X Zhang H Liao S Xu Z. Opsoclonus-myoclonus syndrome associated with herpes simplex virus infection: a case report. Int J Neurosci. (2020) 131:307–11. 10.1080/00207454.2020.1737531 32116082

[24] Shiihara T Takahashi Y. Correspondence: a further case of opsoclonus-myoclonus syndrome associated with Mycoplasma pneumoniae infection. Eur J Pediatr. (2010) 169:639. 10.1007/s00431-009-1105-y 19943062

[25] Huber BM Strozzi S Steinlin M Aebi C Fluri S. Mycoplasma pneumoniae associated opsoclonus-myoclonus syndrome in three cases. Eur J Pediatr. (2010) 169:441–5. 10.1007/s00431-009-1048-3 19774394

[26] Nunes JC Bruscato AM Walz R Lin K. Opsoclonus-myoclonus syndrome associated with Mycoplasma pneumoniae infection in an elderly patient. J Neurol Sci. (2011) 305:147–8. 10.1016/j.jns.2011.03.012 21444093

[27] Mesraoua B Abbas M D'Souza A Miyares FR Hashem M Osman Y. Adult opsoclonus-myoclonus syndrome following Mycoplasma pneumoniae infection with dramatic response to plasmapheresis. Acta Neurol Belg. (2011) 111:136–8. 10.1055/s-0031-1283109 21748933

[28] McCarthy JE Filiano J. Opsoclonus Myoclonus after human papilloma virus vaccine in a pediatric patient. Parkinsonism Relat Disord. (2009) 15:792–4. 10.1016/j.parkreldis.2009.04.002 19447066

[29] Lapenna F Lochi L de Mari M Iliceto G Lamberti P. Post-vaccinic opsoclonus-myoclonus syndrome: a case report. Parkinsonism Relat Disord. (2000) 6:241–2. 10.1016/S1353-8020(00)00020-1 10900400

[30] Wang H. A protocol for investigating the association of vaccination and anti-NMDA receptor encephalitis. Front Biosci (Schol Ed). (2018) 10:229–37. 10.2741/s511 28930529

[31] Mitchell WG Snodgrass SR. Opsoclonus-ataxia due to childhood neural crest tumors: a chronic neurologic syndrome. J Child Neurol. (1990) 5:153–8. 10.1177/088307389000500217 2345282

[32] Brunklaus A Pohl K Zuberi SM de Sousa C. Investigating neuroblastoma in childhood opsoclonus-myoclonus syndrome. Arch Dis Child. (2012) 97:461–3. 10.1136/adc.2010.204792 21460401

[33] de Alarcon PA Matthay KK London WB Naranjo A Tenney SC Panzer JA. Intravenous immunoglobulin with prednisone and risk-adapted chemotherapy for children with opsoclonus myoclonus ataxia syndrome associated with neuroblastoma (ANBL00P3): a randomised, open-label, phase 3 trial. Lancet Child Adolesc Health. (2018) 2:25–34. 10.1016/S2352-4642(17)30130-X 29376112 PMC5783315

[34] Blaes F Dharmalingam B. Childhood opsoclonus-myoclonus syndrome: diagnosis and treatment. Expert Rev Neurother. (2016) 16:641–8. 10.1080/14737175.2016.1176914 27095464

[35] Pranzatelli MR Travelstead AL Tate ED Allison TJ Moticka EJ Franz DN. B- and T-cell markers in opsoclonus-myoclonus syndrome: immunophenotyping of CSF lymphocytes. Neurology. (2004) 62:1526–32. 10.1212/WNL.62.9.1526 15136676

[36] Pranzatelli MR Tate ED Travelstead AL Longee D. Immunologic and clinical responses to rituximab in a child with opsoclonus-myoclonus syndrome. Pediatrics. (2005) 115:e115–9. 10.1542/peds.2004-0845 15601813

[37] Klein A Schmitt B Boltshauser E. Long-term outcome of ten children with opsoclonus-myoclonus syndrome. Eur J Pediatr. (2007) 166:359–63. 10.1007/s00431-006-0247-4 17089089

[38] Goh EL Scarff K Satariano S Lim M Anand G. Evolving cognitive dysfunction in children with neurologically stable opsoclonus-myoclonus syndrome. Children (Basel). (2020) 7:103. 10.3390/children7090103 32824925 PMC7552772

[39] Pranzatelli MR Travelstead AL Tate ED Allison TJ Verhulst SJ. CSF B-cell expansion in opsoclonus-myoclonus syndrome: a biomarker of disease activity. Mov Disord. (2004) 19:770–7. 10.1002/mds.20125 15254934

[40] Pranzatelli MR Slev PR Tate ED Travelstead AL Colliver JA Joseph SA. Cerebrospinal fluid oligoclonal bands in childhood opsoclonus-myoclonus. Pediatr Neurol. (2011) 45:27–33. 10.1016/j.pediatrneurol.2011.02.012 21723456

