# Supplementary material for: Clinical Analysis of Pediatric Opsoclonus-Myoclonus Syndrome in One of the National Children's Medical Center in China
Source: Front Neurol. 2021 Oct 8;12:744041. doi: 10.3389/fneur.2021.744041 (PMC8531251; doi:10.3389/fneur.2021.744041)
Supplement: Supplementary file 1 [file Data_Sheet_1.PDF]

OMS literatures qualified the following requirements were reviewed:1) reported since 2005; 2) more than 20 cases were involved in the study; 3) Treatment were reported; 4) OMS patients both with or without neuroblastoma were included.

Study (published year)/(Number of cases)	age at onset [mean±SD or mean (range)]	tumor(%)	Immunotherapy (%)	Treatment response(%)	Relapse (%)	Neurological sequelae (%)
Tate et al/2005/105(1)	no-tumor :1.6 ± 0.7 y tumor:1.8 ±1.0 y	42	ACTH (52.3), prednisone (47.6), IVIG (41.9), CTX (4.7), AZA (6.7), PELX (1.9)	ACTH (83), prednisone (50), IVIG(50), CTX (<33.3), AZA (<33.3), PELX (<33.3)	52	>75
Krug et al/2010/34(2)	20.8 m (9 m to 6 y)	64.7	Corticosteroid alone (50.0); Corticosteroid with other treatment: + IVIG (17.6), +IVIG + RTX (8.8), + IVIG + CTX (8.8), +unknown (8.8), + IVIG + RTX + CTX (2.9); No treatment (2.9)	NM	41.2	52.9
Tate et al/2012/74(3)	2.0 ±1.4 y	41.9	Corticotropin alone (9.4); Corticotropin +IVIG (23.0); Corticotropin + IVIG + RTX (40.5); Corticotropin +IVIG + CTX (8.1); Corticotropin + IVIG+	≥50% degree of improvement(%): Corticotropin alone (42); Corticotropin + IVIG (70); Corticotropin + IVIG + RTX (83); Corticotropin + IVIG + CTX (83); Corticotropin+IVIG+ RTX + chemotherapy (100); Corticotropin	36	NM

			RTX + chemotherapy (10.8); Corticotropin + IVIG + RTX + steroid parers (8.1)	+ IVIG + RTX +steroid sparers (83); For all group (78)		
Dale et al/2014/32(4)	1.8 y (0.7 y to 8.25 y)	34.4	Immunotherapies Preceding: Steroid (n =30); IVIG (n=30); CTX (n = 10); AZA (n = 1); RTX (n=32)	Probable and definite benefit (75)	NM	68.8
Hasegawa et al/2015/23(5)	16.5 m (11 to 152 m)	43.5	IVIG (73.9), IVMP (56.5), oral prednisolone (52.2), RTX (8.7)	Remission rate: IVIG (35.3), IVMP (23.1), oral prednisolone (33.3), RTX (100)	34.9	73.9
Brunklaus et al/2015/101(6)	18.0 m (3 m to 8.9 y)	21	Steroids (87) including prednisolone (53) and corticotrophin (30); IVIG (11.9); AZA (6.9); CTX (3.0); CsA (2.0); PLEX (2.0)	For all patients: good response (35); Moderate response (60);a slight response (5).	61	66
Pranzatelli et al/2017/389(7)	1.5 y (IQR 1.2–2 y)	50	Steroid only (33); IVIG only (15); Steroid and IVIG only (37); other combinations agents: CTX (8.6), RTX (4.3), AZA	NM	41	NM

			(0.8), MMF (1.3)			
Present study	18 m (10 to 20 m)	55.6	IVIG+IVMP only (55.6) IVIG+IVMP +RTX(44.4)	IVIG+IVMP (100), IVIG+IVMP+RTX (100); RTX reduce relapse rate from 44.4% to 0.0%	44.4	100

NM,not mentioned

Reference

1. E. D. Tate, T. J. Allison, M. R. Pranzatelli and S. J. Verhulst: Neuroepidemiologic trends in 105 US cases of pediatric opsoclonus-myoclonus syndrome. *J Pediatr Oncol Nurs*, 22(1), 8-19 (2005) doi:10.1177/1043454204272560
2. P. Krug, G. Schleiermacher, J. Michon, D. Valteau-Couanet, H. Brisse, M. Peuchmaur, et al.: Opsoclonus-myoclonus in children associated or not with neuroblastoma. *Eur J Paediatr Neurol*, 14(5), 400-9 (2010) doi:10.1016/j.ejpn.2009.12.005
3. E. D. Tate, M. R. Pranzatelli, S. J. Verhulst, S. J. Markwell, D. N. Franz, W. D. Graf, et al.: Active comparator-controlled, rater-blinded study of corticotropin-based immunotherapies for opsoclonus-myoclonus syndrome. *J Child Neurol*, 27(7), 875-84 (2012) doi:10.1177/0883073811428816
4. R. C. Dale, F. Brilot, L. V. Duffy, M. Twilt, A. T. Waldman, S. Narula, et al.: Utility and safety of rituximab in pediatric autoimmune and inflammatory CNS disease. *Neurology*, 83(2), 142-50 (2014) doi:10.1212/wnl.0000000000000570
5. S. Hasegawa, T. Matsushige, M. Kajimoto, H. Inoue, H. Momonaka, M. Oka, et al.: A nationwide survey of opsoclonus-myoclonus syndrome in Japanese children. *Brain Dev*, 37(7), 656-60 (2015) doi:10.1016/j.braindev.2014.10.010
6. A. Brunklaus, K. Pohl, S. M. Zuberi and C. de Sousa: Outcome and prognostic features in opsoclonus-myoclonus syndrome from infancy to adult life. *Pediatrics*, 128(2), e388-94 (2011) doi:10.1542/peds.2010-3114
7. M. R. Pranzatelli, E. D. Tate and N. R. McGee: Demographic, Clinical, and Immunologic Features of 389 Children with Opsoclonus-Myoclonus Syndrome: A Cross-sectional Study. *Front Neurol*, 8, 468 (2017) doi:10.3389/fneur.2017.00468
